# Unraveling Molecular Targets for Neurodegenerative Diseases Through *Caenorhabditis elegans* Models

**DOI:** 10.3390/ijms26073030

**Published:** 2025-03-26

**Authors:** Rongmei Xu, Qiaoju Kang, Xuefei Yang, Ping Yi, Rongying Zhang

**Affiliations:** 1 School of Biotechnology and Food Engineering, Hefei University of Technology, Hefei 230002, China; 2024800090@hfut.edu.cn (R.X.); yangxuefei@hfut.edu.cn (X.Y.); 2 Key Laboratory of Molecular Biophysics of the Ministry of Education, College of Life Science and Technology, Huazhong University of Science and Technology, Wuhan 430074, China; d202080698@hust.edu.cn (Q.K.); pingy@hust.edu.cn (P.Y.)

**Keywords:** *Caenorhabditis elegans*, neurodegenerative disease, model organisms, Alzheimer’s disease, Parkinson’s disease, amyotrophic lateral sclerosis, Huntington’s disease, molecular targets

## Abstract

Neurodegenerative diseases (NDDs), including Alzheimer’s disease (AD), Parkinson’s disease (PD), amyotrophic lateral sclerosis (ALS), Huntington’s disease (HD), and prion disease, represent a group of age-related disorders that pose a growing and formidable challenge to global health. Despite decades of extensive research that has uncovered key genetic factors and biochemical pathways, the precise molecular mechanisms underlying these diseases and effective therapeutic strategies remain elusive. *Caenorhabditis elegans* (*C. elegans*) has emerged as a powerful model organism for studying NDDs due to its unique biological features such as genetic tractability, conserved molecular pathways, and ease of high-throughput screening. This model provides an exceptional platform for identifying molecular targets associated with NDDs and developing novel therapeutic interventions. This review highlights the critical role of *C. elegans* in elucidating the complex molecular mechanisms of human NDDs, with a particular focus on recent advancements and its indispensable contributions to the discovery of molecular targets and therapeutic strategies for these NDDs.

## 1. Introduction

Alzheimer’s disease (AD), Parkinson’s disease (PD), amyotrophic lateral sclerosis (ALS), Huntington’s Disease (HD), and prion diseases are all neurodegenerative diseases (NDDs) characterized by the progressive loss of neurological function [1,2,3,4]. With increasing life expectancy and aging populations, the prevalence of NDDs is escalating, placing substantial economic and healthcare burdens on societies worldwide [1,2]. Although genetic factors associated with these diseases have been identified and numerous therapeutic strategies have been developed, the precise molecular mechanisms governing their progression remain largely unclear. Furthermore, achieving effective treatment and complete recovery remains a formidable challenge in the field [5].

**Figure 1:**
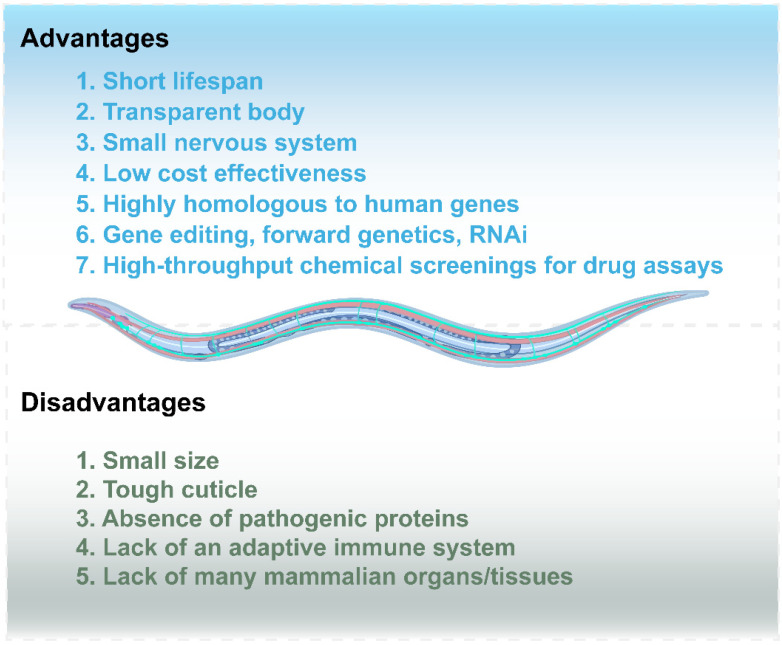
Advantages and disadvantages of *C. elegans* model.

In the pursuit of elucidating NDDs, researchers initially favored invertebrate models such as *Caenorhabditis elegans* (*C. elegans*) over direct experimentation in mammalian systems [6]. Since the 1970s, *C. elegans* has been widely used in neuroscience due to its short life cycle, transparent body, rapid reproduction, and ease of cultivation [7]. These features make it ideal for neuron visualization. Additionally, its large cell size and simple micromanipulation facilitate transgenic model creation, and gene knockout (KO) can be achieved through targeted bacterial feeding [6]. Notably, comparative genomic and proteomic analyses have revealed 83% protein homology and 53% conservation of human protein-coding sequences in *C. elegans*, establishing robust evolutionary relevance for human disease modeling [8,9,10]. Although its nervous system comprises merely 302 neurons, the nematode shares core neurotransmitter systems with mammals, including serotonin, dopamine, acetylcholine, glutamate, and neuropeptide signaling pathways [6,9,10]. Crucially, the biosynthetic regulation, vesicular storage mechanisms, and catabolic processing of these neurotransmitters in *C. elegans* exhibit striking conservation with mammalian counterparts (Figure 1) [11,12]. In a word, these synergistic advantages collectively position *C. elegans* as a premier experimental platform for decoding NDD pathogenesis and validating therapeutic targets [13,14].

This review aims to systematically overview the current research models of NDDs, such as AD, PD, ALS, and HD, using nematode models. It also seeks to summarize the key molecular targets associated with these NDDs. In summary, this review aspires to provide valuable theoretical and practical insights into utilizing nematode models to advance research and development of therapeutic strategies for NDDs.

## 2. Alzheimer’s Disease (AD)

AD is a devastating neurodegenerative disorder, ranking sixth among leading causes of death. By 2060, it is estimated that 13.8 million people will be affected [15,16]. AD has two primary forms: early-onset AD (EOAD) and late-onset AD (LOAD), with the latter comprising over 95% of cases, typically emerging after age 65 [17]. Clinically, AD manifests as cognitive decline, memory impairment, and neuropsychiatric symptoms. Pathologically, it is characterized by amyloid-beta plaques and neurofibrillary tangles (NFTs) from tau protein aggregation in neurons [18]. Ethical considerations compel researchers to turn to animal models such as *C. elegans*, capable of expressing human-related genes, to unravel the potential mechanisms and molecular targets underlying AD [19].

### 2.1. Amyloid-Beta Peptide (Aβ Peptide)

The initial *C. elegans* model of AD was created by overexpressing (OE) the Aβ peptide, derived from the amyloid-beta precursor protein [20]. Under normal conditions, Aβ production and degradation are balanced, but this balance is disrupted in AD, leading to elevated Aβ levels [21]. Two major Aβ isoforms, Aβ1–40 and Aβ1–42, have been identified in the brains of AD patients [22]. Since *C. elegans* lacks the enzyme to produce Aβ, transgenic models have been developed to express human Aβ in specific cells [23]. Notably, Aβ1–42 is more prone to forming oligomers and exhibits greater neurotoxicity, making it a key focus for AD research and drug screening [24].

Research on molecular targets to mitigate Aβ toxicity primarily focuses on several critical areas: enzyme activities, protein oxidative factors, molecular chaperones, and alterations in the Hippo and autophagy-lysosome pathways. Studies utilizing *C. elegans* models have yielded important insights. Firstly, the absence of O-GlcNAc transferase (OGT) alleviated Aβ-induced damage, suggesting OGT as a potential therapeutic target for AD. Conversely, the loss of β-N-acetylglucosamine glycosidase exacerbated Aβ-induced damage [25]. Secondly, protein oxidation significantly contributes to Aβ1–42-induced neurodegeneration. In nematodes, the deletion of the eEF2K homolog efk-1 mitigated oxidative stress and reduced Aβ1–42 toxicity [26]. Thirdly, the molecular chaperone HSP-16.2 also offers protection against Aβ-induced toxicity. Consistent with this notion, heat shock treatment in transgenic nematodes reduced Aβ toxicity [27]. Notably, overexpression of HSP-16 has been reported to suppress the toxicity of GFP::degron, a system that recapitulates Aβ aggregate-induced pathology [28]. Furthermore, the interplay between the Hippo and mTOR signaling pathways promotes AD development in *C. elegans* Aβ models by increasing Aβ peptide production. Aβ cytotoxicity is negatively regulated by the CST-1/SAV-1/MOG-1/WTS-1 cascade and positively regulated by the YAP-1/EGL-44 co-transcriptional complex [29]. LIN-10, a homolog of mammalian MINS3 in *C. elegans*, interacts with YAP-1 to influence its nuclear translocation. Knockdown of lin-10 mitigated Aβ accumulation. Moreover, Florez-Mcclure et al. revealed that impaired lysosomal function exacerbates Aβ toxicity and triggers autophagy aggregation. Their research demonstrated that using RNAi to knock down genes encoding lysosomal components (including aspartic proteases (*asp-2*, *asp-4*, *asp-5*, and *asp-6*), lysosomal-associated membrane proteins (*lmp-1* and *lmp-2*), and vacuolar proton translocation ATPase (*vha-15*)) led to increased paralysis rates and higher Aβ1–42 levels. As a result, it is proposed that Aβ1–42 toxicity in *C. elegans* may arise from defective lysosome formation or impaired degradation [30]. Collectively, these findings highlight the potential of enhancing the Hippo signaling pathway and the autophagy-lysosome pathway as promising therapeutic or preventive strategies for AD.

### 2.2. Tau Protein

Tau protein is a critical biomarker for AD, essential for stabilizing neuronal microtubules. In *C. elegans*, the tau-like repeat protein PTL-1 (the sole homolog of both tau and microtubule-associated protein 2 (MAP2)) plays a regulatory role in neuronal aging and lifespan [31,32]. Transgenic models expressing human tau exhibit age-related motor neuron impairments and neurodegenerative changes [33]. Mutated tau forms linked to insoluble phosphorylated aggregates and motor deficits have been explored [34,35]. Significantly, in addition to the correlation between phosphorylation at specific sites of the tau protein and AD, acetylation at specific sites has also been demonstrated to play a crucial role in the progression of tau pathology [36,37,38,39,40]. Genetic editing advancements have enhanced our understanding of tau homeostasis in *C. elegans* [41,42]. Notably, a dual transgenic nematode model incorporating both Aβ1–42 and tau genes closely mimics the neuropathological features of AD [43].

Scientists are focusing on identifying genetic factors that affect tau-induced neurotoxicity. Forward genetic screens have revealed mutations that reduce tau-induced problems. For example, the recessive mutation *sut-1*(*bk79*) partially lessened uncoordinated movement, tau aggregation, and neurodegenerative changes [44]. OE *sut-2* worsened neuronal dysfunction and increased tau accumulation, while mutations that reduced its function protect against lifespan shortening [45,46]. Losing the RNA binding function of SUT-6 or reducing its expression has been demonstrated to suppress the phenotype associated with tau pathology in nematodes expressing human tau protein [47]. The deletion of ALYREF, a protein involved in mRNA splicing, reduced toxicity in tau and TDP-43 neurodegeneration models. Although there are three genes of ALYREF (*aly-1*, *aly-2*, and *aly-3*) in *C. elegans*, it is noteworthy that only the loss of function of aly-2 and aly-3 can alleviate tau-induced toxicity [48]. Similarly, reducing the function of the SPOP-1 protein improved behavioral deficits in tau transgenic nematodes, while overexpressing it worsened these deficits [49]. Recent studies have shown that reducing DNA glycosylases NTH-1 or UNG-1 enhances mitochondrial function, lifespan, and memory in tau transgenic nematode models. And the endoplasmic reticulum unfolded protein response (UPRER) transcription factor XBP-1s helped clear pathological tau through the ATF-6 branch of UPRER [50]. In summary, research into AD using Tau transgenic nematode models has revealed genetic factors and molecular targets involved in tau toxicity, identifying potential therapeutic targets for future interventions.

## 3. Parkinson’s Disease (PD)

PD is the second most common NDD, characterized by movement impairments and the loss of dopaminergic neurons [51]. Alpha-synuclein (alpha-Syn) mutations are a key cause of hereditary PD [52,53]. Experimental animal models are essential in PD research, and they can be categorized into two primary types, transgenic models and neurotoxin-induced models [54,55]. The nematode not only possesses a simple nervous system but also shows high homology between its genes and human PD-related genes. These characteristics make it an ideal model organism for PD research [56]. Recent research has made significant progress using nematode models to understand PD [54,56]. In this review, we will expound several extensively examined nematode models of PD.

### 3.1. Transgenic Model

#### 3.1.1. Alpha-Synuclein (Alpha-Syn)

The alpha-Syn protein, composed of 140 amino acids, is encoded by the human SNCA gene linked to autosomal dominant PD [57,58,59]. However, nematodes are valuable PD models as they have many homologous genes to human PD-associated genes, excluding *park1*, which encodes alpha-Syn. Consequently, allowing expression of human alpha-Syn without endogenous interference [55,60]. Garry Wong’s team pioneered transgenic *C. elegans* models as early as 2003, including wild-type and A53T human alpha-Syn models that are specifically expressed in dopaminergic neurons or pan-neurons [61]. Notably, the transparency of *C. elegans* enables in vivo visualization of dopamine (DA) neuron morphology and neurodegeneration when co-injected with green fluorescent protein (GFP) [61]. Long-term studies of this model have identified proteins like calmodulin, RAB1, RAB3, RAB8, RAB10, RHO, ATP13A2, VPS-41, cathepsin D, GPI, GBA, ATFS-1, and 14–3-3 as regulators of DA neuron neurodegeneration, with similar effects observed in mammalian models [62,63,64,65,66,67,68,69].

In addition to the molecular targets that regulate toxicity, as aforementioned, there have also been studies emphasizing the protective effects of developmental regulatory factors, autophagy, and small RNAs in neurons against toxicity [62,70,71]. The *C. elegans* model with TOR-2 and alpha-Syn-GFP provides valuable insights into genetic modifiers of alpha-Syn misfolding [62]. Furthermore, Tyson et al. employed a bimolecular fluorescence complementation (BiFC)-alpha-Syn nematode model to investigate the mechanism underlying alpha-Syn transfer between neurons. They observed that RNAi-mediated inhibition of autophagy-related genes led to a decrease in BiFC-induced fluorescence, highlighting the role of autophagy in the clearance and dissemination of alpha-Syn [72]. Genetic studies have yielded significant insights into the role of microRNAs (miRNAs) in PD, particularly through *let-7* miRNA regulation. Shamsuzzama et al. investigated the role of *let-7* miRNA in PD using a transgenic *C. elegans* model expressing “human” alpha-Syn [73,74,75]. Their study demonstrated that while *let-7* miRNA modulates PD-associated pathways through autophagy enhancement and DAF-16 FOXO transcription factor activation-mediated downregulation of alpha-Syn expression, it exhibited no detectable effects on dopaminergic/acetylcholinergic neurons. Collectively, these findings highlight *let-7* miRNA as a potential novel therapeutic target for PD intervention [75]. Additionally, gene product RTCB-1 and the recently discovered RNA editing enzyme ADR-2 have also been shown to alleviate alpha-Syn toxicity [73,74]. In summary, there are various regulatory factors targeting alpha-Syn toxicity, such as cell transport pathways, autophagy pathways, neuronal development regulatory factors, and small RNA molecules.

#### 3.1.2. Leucine-Rich Repeat Kinase 2 (LRRK2)

LRRK2 is a ROCO protein that plays a pivotal role in membrane trafficking and synaptic vesicle endocytosis [76,77]. Its nematode homolog LRK-1 is widely expressed [78]. LRRK2 mutations cause familial PD, making it a therapeutic target [79]. The utilization of LRRK2 KO and transgenic OE models has been documented in *C. elegans*, such as the OE of human LRRK2 G2019S and R1441C in *C. elegans*, which has been demonstrated to induce DA neuron neurodegeneration, leading to a decrease in DA levels and impairment of motor function [80,81]. Notably, the LRRK2 kinase inhibitors TTT-3002 and LRRK2-IN1 exhibited neuroprotective effects in transgenic *C. elegans* models of OE human R1441C and G2019S LRRK2 mutations [82]. Moreover, the prevalent G2019S mutation exhibits a particularly strong association with alpha-Syn pathology. Early work in 2015 demonstrated that, unlike WT LRRK2, which enhances autophagic flux throughout the *C. elegans* lifespan, the G2019S LRRK2 mutation suppresses autophagy and accelerates age-related autophagic decline. Critically, the G2019S LRRK2 mutation synergizes with alpha-Syn to exacerbate autophagy inhibition in this model [83]. Recent studies further revealed that the G2019S LRRK2 mutation not only boosts LRRK2 kinase activity but also plays a pivotal role in the replication of alpha-Syn [84,85,86]. Further study revealed that LRRK2 controls alpha-Syn replication via RAB35 phosphorylation [86]. These results indicate a complex regulatory relationship between PD-related genes.

Although oxidative stress is linked to PD pathology, the connection between LRRK2 G2019S and stress in PD remains unclear. Ray et al. showed that LRRK2 G2019S increases sensitivity to stress and impairs DAF-16 nuclear translocation, a key factor for stress resistance in *C. elegans* [87]. As we all know, enhancing DAF-16 extends the lifespan of LRRK2 G2019S mutants and reduces neuronal damage, while down-regulating it has the opposite effect. Further research found that DAF-16 may also reduce LRRK2 G2019S toxicity via 14–3-3 proteins [88,89,90,91]. Moreover, another study revealed that Glutaredoxin 1 (Grx1), an important antioxidant enzyme, significantly exacerbates the neurodegenerative phenotype of LRRK2 mutant G2019S or R1441C OE in *C. elegans* [92]. Lately, studies have revealed that various pathways involved in aging and longevity, such as insulin/IGF-1 signaling, the TOR pathway, and mitochondrial respiration, exhibit robust protective effects against LRRK2 G2019S-induced neurodegeneration [85]. Coincidentally, recent studies have shown that overexpression of miRNA-71 (regulating modulates stress resistance and promotes longevity in *C. elegans*) can rescue motor deficits and attenuate DA neuron degeneration, highlighting its role in mitigating LRRK2-induced proteotoxicity [93,94]. These findings suggest that oxidative stress and longevity regulators represent critical molecular targets for the development of LRRK2-targeted PD therapies.

### 3.2. Toxin-Induced Models-6-Hydroxydopamine (6-OHDA)

As is well known, there are various sources of toxins that can induce PD, among which the most common is the hydroxylated form of DA, known as 6-OHDA. It is a specific catecholaminergic neurotoxin widely employed for the induction of PD-like features [95]. 6-OHDA binds to dopamine transporters (DAT) to enter nigrostriatal dopamine neurons, but it cannot cross the blood-brain barrier. Subsequently, it undergoes multiple oxidation processes leading to cellular damage [96]. In a previous study conducted by Harrington A J et al., it was demonstrated that using transgenic technology, fluorescent reporter genes (e.g., GFP) driven by neuron-specific promoters (such as dat-1 or rab-3) were integrated into the *C. elegans* genome, enabling direct observation of neuronal apoptosis or other abnormal changes [97]. Treatment with 6-OHDA in a specific strain resulted in time- and dose-dependent DA neuron degeneration, reducing GFP spots. Numerous recent studies have also shown that 6-OHDA-induced dopaminergic neurodegeneration in *C. elegans* [98,99,100,101].

6-OHDA uptake by neurons generates free radicals and oxidative stress, leading to neuronal death, suggesting that oxidative stress regulatory factors may be an important molecular target for treating 6-OHDA-induced PD. As expected, *skn-1*, a gene that boosts oxidative stress resistance and lifespan in nematodes, has been found to enhance their protection against 6-OHDA-induced neurodegeneration through excessive activation [101]. Not only that, this study also showed that mutations in calchaperonin *crt-1*, *tsp-17*, and *gilt-1* prevent 6-OHDA-induced PD-like phenotype [102]. Additionally, the *ttr-33* gene in *C. elegans* offered protection against 6-OHDA-induced oxidative stress [103]. Through continuous research, it has been discovered that antioxidant compounds like Aureusidin, Tricetin, and Galangin protect against 6-OHDA-induced neurodegeneration in PD models via the Nrf2 pathway [104,105,106]. Recently, a high-sugar diet in adulthood was found to protect *C. elegans* from 6-OHDA-induced DA neurodegeneration by altering redox state, not ATP levels [107]. To sum up, oxidative stress regulators are highly potential therapeutic targets for 6-OHDA-induced PD.

## 4. Amyotrophic Lateral Sclerosis (ALS)

ALS is a severe neurodegenerative disease affecting about one in 350 individuals [108,109]. It is characterized by motor neuron degeneration in the cerebral cortex, brain stem, and spinal cord. Most cases (>90%) are sporadic ALS (sALS) without clear genetic factors, while 5–10% are familial ALS (fALS) due to inherited mutations [110]. Fortunately, over 45 human genes are involved in ALS, including *Cu/Zn superoxide dismutase 1* (*SOD1*), *TAR DNA-binding protein 43* (*TDP-43*), *Chromosome 9 Open Reading Frame 72* (*C9orf72*), and *Fusion sarcoma* (*FUS*) [111]. Significant progress has been made in understanding the pathogenic mechanisms of these four genes in ALS using *C. elegans* transgenic models [112].

### 4.1. Cu/Zn Superoxide Dismutase 1(SOD1)

The first gene associated with ALS is *sod1*. SOD1 is inherited in an autosomal dominant manner and contains over 180 missense mutations, accounting for 10–14% of fALS cases and 1–2% of sALS cases [113,114,115,116,117]. Notably, disease severity and survival time vary based on mutation sites, and Cys residues in the SOD1 protein are crucial for its pathogenicity [118]. The nematode model of ALS was first established in 2001, revealing that SOD1-induced motor neurotoxicity involves protein misfolding and disruptions in cellular protein homeostasis, axonal development, ER stress, and autophagy [119,120]. The investigation into the major mutant G85R of human SOD1 (hmSOD1) revealed that its neurotoxic effects are associated with SOD1 misfolding and synaptic vesicle disruption, thereby providing further evidence for the toxic mechanisms induced by SOD1 as previously discussed. Expectedly, using this model found that the DAF-2 insulin/IGF-1 signaling pathway has therapeutic potential for ALS; for instance, downregulation of DAF-2 signaling or silencing of cytokinin homologs *grp1* or *efa-6* was observed to significantly reduce SOD1 aggregation and improve movement defects in hmSOD1 mutant G85R nematodes [121]. Additionally, the integrative treatment of the hmSOD1 mutant G85R/G93A nematode model with DAF-2 further confirmed the potential therapeutic efficacy of DAF-2 insulin/IGF-1 signaling in ALS [122]. In addition to the involvement of aging control factors in the neuroprotective effects of SOD1 transgenic nematodes, it has also been reported that BTBD10 (the product of the human DYT1 gene torsinA), a ubiquitin-specific protease USP7, a valine-containing protein (VCP), and RAD-23 (a component of the protein homeostasis network and nucleotide excision repair pathway) have different degrees of protection against SOD1-induced cytotoxicity [123,124]. These aforementioned results highlight that the regulation of SOD1 protein homeostasis is a pivotal research avenue for investigating molecular targets implicated in SOD1-induced ALS.

### 4.2. TAR DNA-Binding Protein 43 (TDP-43)

TDP-43 is a conserved RNA/DNA-binding protein associated with ALS and FTD, characterized by cytoplasmic inclusions [125,126]. It is encoded by the TARDBP gene, and so far over 50 TARDBP gene mutations have been associated with ALS. TDP-43 consists of four main domains, among which the c-terminus is prone to aggregation. However, its homolog TDP-1 in *C. elegans* lacks the glycine-rich c-terminus domain [127,128]. Research has found that the neurotoxic mechanisms of TDP-43 and TDP-1 in nematodes involve the insulin/IGF signaling pathway [127,128,129,130]. In addition, human TDP-43 can repair defects in *C. elegans* lacking TDP-1 [131]. These above results indicate that TDP-43 and TDP-1 are functionally conserved. Notably, a pivotal investigation revealed that granulin (a proteolytic cleavage product of progranulin) exacerbates TDP-43 neurotoxicity via two distinct mechanisms: (1) promoting cytoplasmic aggregation of TDP-43 and (2) increasing its total cellular abundance through impaired autophagic-lysosomal degradation [131].

TDP-43 mutants, especially mutants TDP-43 (A315T)/(M337V)/C-terminus, facilitate the identification of genes associated with neurodegenerative disorders. In *C. elegans* expressing TDP-43 (A315T), losing ALYREF function in neurons improved motor function [48]. Similarly, TIR-1 pathway gene inactivation provided protection for TDP-43 (A315T). Especially neurosecretory gene (*unc-13*(*e540*)/UNC13C and *unc-31(e928*)/CADPS2) mutations not only rescued neurodegeneration but also indicated that neurosecretion is crucial for inducing innate immunity in *C. elegans* with TDP-43 (A315T) [132]. Furthermore, the *tir-1*(*qd4*) deletion reduced immune responses and motor deficits without altering TDP-43 levels [132]. Studies on the neurotoxicity induced by specific expression of TDP-43 (A315T) in GABAergic motor neurons emphasized the importance of ER calcium-regulated enzymes in neurotoxicity [133]. Since TDP-43 hyperphosphorylation disrupts protein homeostasis, leading to neurotoxicity, identifying a TDP-43-specific phosphokinase is a potential intervention target. [134]. A CDC7 kinase inhibitor was identified in *C. elegans* with mutant TDP-43 (A315T), and PRKD2/3 and TTBK1/2 were found to phosphorylate TDP-43 [134,135,136]. However, only the neuronal expression of TTBK1 worsened motor deficits and increased TDP-43 phosphorylation and aggregation [134,135,136]. Excitingly, ethylsulfonimide-based screening revealed α-methyl-α-phenylsuccinimide (MPS) improved motor issues and rescued neuron degeneration in *C. elegans* with mutant TDP-43 (A315T) [137]. For TDP-43 (M337V), RNAi screening found *hse-5*, *zig-3*, *paqr-1*, *gly-8*, and *sax-2* as motor defect modifiers. Mutations in these genes reduced TDP-43 accumulation and phosphorylation [130,138]. Notably, RAD23 serves as a common regulatory factor for both SOD1 and TDP-43, and functional mutations in RAD-23 have been found to rescue phenotypes such as motor defects, GABAergic motor neuron degeneration, and TDP-43 (M337V) aggregation [124]. The latest research findings indicated that lower temperatures extended lifespan and mitigated TDP-43 (M337V) accumulation in nematodes. However, inhibition of proteasome regulators *psme-3*/PSME3 has been shown to impede the reduction of TDP-43 protein aggregates [139]. For C-terminal mutants of TDP-43, overexpression solely in neurons led to aggregation and motor defects, but this situation can be fully ameliorated by deleting SPR5/LSD1 and UFD-2/UBE4B [140]. To reduce TDP-43-C-terminal-induced neurotoxicity, PROTACs have been designed to degrade misfolded C-TDP-43 proteins recently. PROTAC2 effectively decreases TDP-43-C-terminal accumulation in the nervous system, improving motor function in *C. elegans* [141]. Thanks to the development of genetic manipulation techniques, another small molecule, TRVA242, also showed neuroprotective effects in TDP-43 nematode and zebrafish ALS models [142]. Overall, the functional mutations of the aforementioned factors were found to be capable of effectively reducing the accumulation and pathological phosphorylation of TDP-43. Thus, they are highly potential molecular targets for the treatment of amyotrophic lateral sclerosis.

### 4.3. Chromosome 9 Open Reading Frame 72 (C9orf72)

The C9orf72 gene on chromosome 9 encodes a protein crucial for autophagy regulation and GTPase activation [143,144,145]. ALS/FTD pathogenesis often involves GGGGCC (G4C2) repeat expansion in its first intron, predominantly in European ALS patients [146,147]. Normally, G4C2 repeats are less than 30, but in fALS, repeats can exceed hundreds [148]. C9orf72-related ALS/FTLD involves loss of function, RNA toxicity, and toxic dipeptide protein accumulation. Deleting the gene caused paralysis, nuclear transport issues, lysosomal dysregulation, and neurodegeneration [149,150,151]. Studies on C9orf72 homolog alfa-1 in *C. elegans* revealed age-related motor defects and stress sensitivity in alfa-1 mutants [151]. Anna Corrionero et al. discovered that the expression of the C9orf72 protein in *C. elegans* partially rescued the spot-like phenotype caused by the alfa-1 deletion mutation, suggesting a functional conservation between C9orf72 and ALFA-1 [150]. Moreover, both ALFA-1 and C9orf72 interact with Rag and Rab-GTPases, affecting mTOR signaling and endosomal trafficking [152,153,154]. Notably, alfa-1 deletion exacerbated motor impairments from TDP-43 toxicity, indicating complex ALS gene interactions [151].

Through forward genetic screening, F57A10.2/VAMP and lysosomal acid phosphatase ACP-4/ACP2 were identified as suppressors of C9orf72-related phenotypes [149]. Furthermore, the nuclear E3 ubiquitin ligase linker protein SPOP and the eukaryotic translation initiation factor 2D (eIF-2D/eIF2D) can inhibit the protein toxicity of PR50 (Pro Arg 50, a dipeptide repeat protein (DPR)), indicating that they are therapeutic targets for C9orf72-related ALS/FTD [155,156]. Notably, another study showed that deletion of Lethal (3) malignant brain tumor-like protein 1 (L3MBTL1) effectively reduces the toxicity associated with both SOD1 and C9orf72 mutations [157]. These modifiers suggest autophagy, the ubiquitin proteasome system, RAN translation, and stress granules as potential therapeutic targets for C9orf72-related ALS.

### 4.4. Fusion Sarcoma (FUS)

FUS is a 526-amino acid RNA-binding protein that is similar to TDP-43 and predominantly localizes within the nucleus but can shuttle between the nucleus and cytoplasm [158,159,160,161]. To date, more than 100 pathogenic FUS variants have been identified in ALS patients. Most of them are missense mutations, and the pathogenic mutations are mainly located in the C-terminal, RGG binding, or nuclear localization signal (NLS) domains of the protein, which disrupt FUS localization, transcription, RNA maturation, and form toxic aggregates [161,162,163,164,165,166]. Pathogenic *fus* gene variants, often inherited dominantly, are linked to early-onset ALS [167]. In addition to ALS, cytoplasmic FUS aggregations are also found in other NDDs, suggesting a crucial role in these diseases [168]. The FUS homolog FUST-1 in *C. elegans* shares similarities with FUS [169,170,171]. Deletion of fust-1 leads to ALS-like phenotypes [172]. A humanized ALS model using fust-1 mutant *C. elegans* revealed the role of FUST-1 in modulating SOD-1, VGLUT/EAT-4, GLR-1, and oxidative stress, offering insights into ALS pathogenesis and therapeutic targets [173].

Studies in *C. elegans* show that FUS mutants cause neuronal dysfunction and death, whereas WT-FUS overexpression does not induce ALS-like phenotypes. ALS cases with FUS mutations, especially the R495X mutant, exhibit early-onset motor neuron damage and rapid neurodegeneration [161]. C-terminal NLS mutants of FUS, like FUS501, shorten lifespan and impair motor function [174]. Moreover, single-copy transgenic *C. elegans* expressing human FUS in GABAergic neurons display ALS-like phenotypes [168]. Furthermore, CRISPR-Cas9 created R524S and P525L ALS FUS models in *C. elegans*, which show neuromuscular and motor impairments under stress, dependent on SQST-1 [172]. Recently, one study suggested that the P525L mutation is particularly aggressive, affecting stress granule dynamics and enhancing interaction with PARP1 (an enzyme responsible for catalyzing poly-ADP-ribosylation) in human iPSC-derived motor neurons. When knocking down the *C. elegans*, histone H1.2 and PARP1 homologues significantly reduced the FUSP525L aggregation and neurodegeneration [175]. Despite significant advancements in generating FUS related mutant *C. elegans* models for neurodegeneration research, a striking knowledge gap persists in mapping their druggable molecular targets, particularly within context-dependent proteostasis networks.

## 5. Huntington’s Disease (HD)

HD is a rare autosomal dominant neurodegenerative disorder caused by CAG trinucleotide repeat expansion in the huntingtin (HTT) gene, leading to aggregation of polyglutamine (polyQ)-expanded mutant huntingtin protein [6,176,177,178,179]. *C. elegans* models (e.g., Htn-Q150, Q40::YFP, or Q128::GFP) expressing polyQ peptides in sensory neurons recapitulate key pathological features, including progressive protein aggregation and neurodegeneration [180,181]. These models have identified three classes of conserved neuroprotective targets, (1) Protein homeostasis regulators—molecular chaperones (HSP-70/-40/-90), autophagy components (*atg-1*, *lgg-1*), HSF-1 transcription factor, and insulin/IGF-1 signaling (*daf-2*/*daf-16*) mitigate polyQ toxicity by enhancing refolding, degradation, or clearance of misfolded proteins [182,183,184,185,186]; (2) Post-translational modifiers—deacetylases (Sirtuin-1/2), ubiquitination-related proteins (UBR5, UBC-25, RPN-6, and AMPK), and phosphorylation-associated proteins. TBK1 regulates protein quality control [186,187,188,189,190,191,192]; (3) Mitochondrial homeostasis factors—such as *pgp-3* genes and mitochondrial fission suppressors ameliorate locomotor defects via RNAi screens [193,194,195,196]. Pharmacological interventions (e.g., rapamycin) further rescue motor deficits by inducing autophagy [197].

Despite these advances, translational challenges persist. The absence of endogenous HTT in *C. elegans* limits exploration of context-dependent polyQ toxicity (e.g., nuclear aggregation effects) [198]. Species-specific outcomes—such as HSP-90 inhibition alleviating toxicity in worms but exacerbating neurodegeneration in Drosophila—highlight divergent chaperone functions [199]. Moreover, protective effects of autophagy induction (e.g., rapamycin) and chaperone modulation show inconsistent efficacy in mammalian models, underscoring the need for cross-species validation and models expressing full-length mutant HTT to bridge mechanistic insights to therapeutic development [185,200,201].

## 6. Prion Diseases

Prion diseases, also known as transmissible spongiform encephalopathies (TSEs), are a group of fatal neurodegenerative disorders caused by the misfolding of the cellular prion protein (PrP^C^) into a pathogenic isoform (PrP^Sc^) [4]. This conformational change triggers self-propagating aggregation, leading to neuronal loss, spongiform vacuolation, and gliosis in the brain. Unlike other neurodegenerative diseases, prion diseases exhibit infectious, sporadic, and genetic origins, with clinical manifestations including rapid cognitive decline, ataxia, and myoclonus [4]. The use of *C. elegans* to model prion diseases has provided valuable insights into the mechanisms of prion propagation and toxicity. By manipulating genes involved in protein quality control and cellular stress responses in *C. elegans*, researchers have been able to identify potential therapeutic targets for prion diseases [202,203,204].

High-throughput screening in *C. elegans* has uncovered key regulatory networks in prion pathogenesis. Firstly, dual roles of chaperone systems: HSP-70 family members (e.g., HSP-1) delay PrP aggregation through direct binding, but excessive activation exacerbates toxicity, indicating dose-dependent regulation [205]. Secondly, modulation of autophagy-lysosome pathways: RNAi screens reveal that knockdown of *atg-5* or *lgg-1* (LC3 homolog) increases PrP aggregation burden, while rapamycin-induced autophagy alleviates motor deficits [206]. Thirdly, cross-species conserved synergistic toxicity: PrP and HD-associated polyQ proteins exhibit cooperative aggregation in *C. elegans*, suggesting shared pathogenic pathways among misfolded proteins [195]. Moreover, CRISPR-based gene editing knockout of PrP receptor homologs (e.g., LAMR-1, a laminin receptor analog) inhibits PrP endocytosis and cell-to-cell propagation [196]. Although *C. elegans* models show promise in prion disease research, several challenges remain [203]. For example, the absence of endogenous PrP genes in worms may hinder accurate recapitulation of native mammalian PrP conformational dynamics; current models struggle to replicate PrP’s trans-synaptic spread within the central nervous system. Future studies could address these gaps by developing humanized PrP transgenic strains, integrating microfluidic chips to simulate neural circuits, and applying single-cell sequencing to resolve spatiotemporal propagation patterns of prion proteins.

## 7. Discussion and Future Perspectives

**Figure 2:**
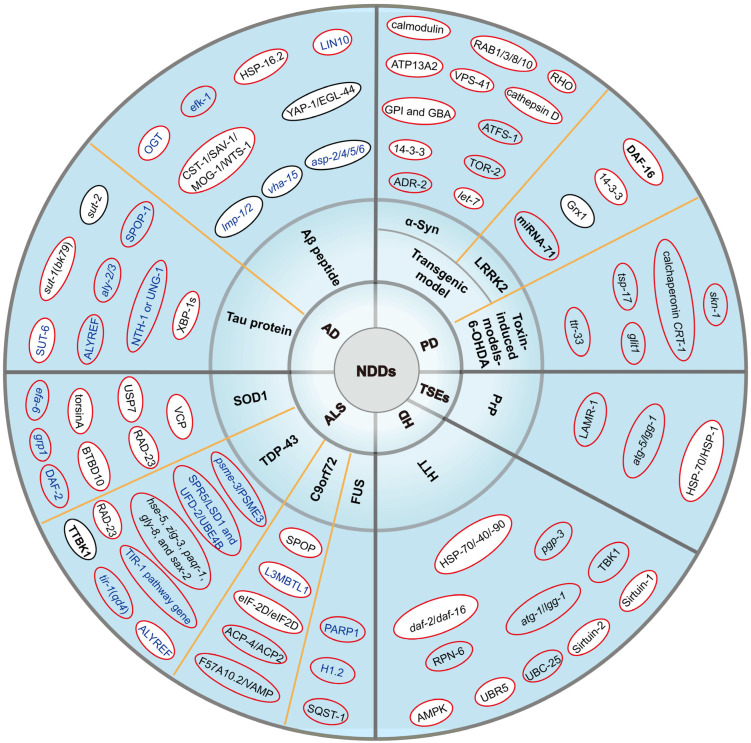
The pie chart illustrated the primary molecular targets associated with AD, PD, ALS, and HD, respectively. Red circle: positive regulation; Black circle: negative regulation; Blue font: gene deletion/downregulation/inactivation; Bold font: overexpression/activation. White background: the molecular targets that have also been supported in mammalian studies.

*C.elegans* has emerged as a cornerstone model for deciphering molecular mechanisms and targets underlying NDDs, leveraging its genetic tractability, conserved pathways, and capacity for high-throughput screening (Figure 2). Studies across AD, PD, ALS, HD, and prion diseases have consistently highlighted the centrality of proteostasis networks in mitigating protein misfolding and aggregation. For example, molecular chaperones (e.g., HSP-70/HSP-1) exhibit dual roles in polyQ and PrP toxicity, where moderate activity suppresses aggregation but chronic overexpression exacerbates proteostatic stress [182,205]. Similarly, autophagy-lysosome pathways and ubiquitin-proteasome systems are critical for clearing toxic species like Aβ, alpha-Syn and TDP-43, as demonstrated by rapamycin-induced autophagy rescuing motor deficits in HD and PD models [101,197]. The nematode’s transparent body and mapped nervous system further enable real-time tracking of neurodegeneration, such as Thioflavin T-positive Aβ plaques in AD and synaptic vesicle mislocalization in PD [24,61]. Despite these advances, translational challenges persist. The absence of endogenous human disease genes (e.g., HTT and PrP, etc.) limits the recapitulation of nuclear aggregation or trans-synaptic prion spread [196,198], while species-specific discrepancies (such as HSP-90 inhibition alleviating toxicity in worms but worsening neurodegeneration in Drosophila) underscore the need for cross-validation [199]. Moreover, inconsistent efficacy of autophagy induction in mammalian models emphasizes the complexity of scaling nematode-derived insights to humans [185,200].

To bridge these gaps, future research should prioritize three frontiers: (1) Model refinement—leveraging CRISPR-Cas9 for polygenic NDD modeling, humanized transgene integration (e.g., full-length HTT or C9orf72 repeats), and organoid-coculture systems to mimic neuronal-glial crosstalk; (2) Multi-omics integration—applying single-cell transcriptomics and spatially resolved proteomics to resolve neuron-specific vulnerability and spatiotemporal aggregation dynamics; and (3) Translational innovation—developing in vivo high-content screens for small-molecule chaperone inducers and CRISPR-based gene therapies targeting aging-modulated pathways (e.g., daf-2/daf-16). Additionally, exploring the synergy between proteostasis enhancers and anti-aging interventions (e.g., mitochondrial UPR activators) could unveil combinatorial therapies. Finally, validating *C. elegans*-derived targets in patient-derived iPSC models or 3D bioprinted neural tissues will bridge the gap between nematode genetics and clinical applicability. By addressing these challenges, *C. elegans* will remain indispensable for decoding NDD complexity and accelerating therapeutic discovery.

## Data Availability

No new data were created or analyzed in this study. Data sharing is not applicable to this article.

## Abbreviations

The following abbreviations are used in this manuscript:

NDDs	Neurodegenerative diseases
AD	Alzheimer’s Disease
PD	Parkinson’s Disease
ALS	amyotrophic lateral sclerosis
HD	Huntington’s Disease
*C. elegans*	*Caenorhabditis elegans*
KO	knockout
EOAD	early-onset AD
LOAD	late-onset AD
NFTs	neurofibrillary tangles
OE	overexpressing
Aβ peptide	amyloid-beta peptide
OGT	O-GlcNAc transferase
MAP2	microtubule-associated protein 2
UPRER	endoplasmic reticulum unfolded protein response
alpha-Syn	alpha Synuclein
DA	dopamine
GFP	green fluorescent protein
BiFC	bimolecular fluorescence complementation
miRNAs	microRNAs
LRRK2	Leucine-rich repeat kinase 2
Grx1	Glutaredoxin 1
6-OHDA	6-hydroxydopamine
DAT	dopamine transporters
PQ	Paraquat
sALS	sporadic ALS
fALS	familial ALS
SOD1	Cu/Zn superoxide dismutase 1
TDP-43	TAR DNA-binding protein 43
C9orf72	Chromosome 9 Open Reading Frame 72
FUS	Fusion sarcoma
hmSOD1	human SOD1
VCP	valine-containing protein
MPS	α-methyl-α-phenylsuccinimide
DPRs	dipeptide repeat protein
L3MBTL1	Lethal (3) malignant brain tumor-like protein 1
G4C2	GGGGCC
NLS	nuclear localization signal
HTT	huntingtin
polyQ	polyglutamine
TSEs	transmissible spongiform encephalopathies
PrP	prion protein
